# NSABP FB-10: a phase Ib/II trial evaluating ado-trastuzumab emtansine (T-DM1) with neratinib in women with metastatic HER2-positive breast cancer

**DOI:** 10.1186/s13058-024-01823-8

**Published:** 2024-04-22

**Authors:** Samuel A. Jacobs, Ying Wang, Jame Abraham, Huichen Feng, Alberto J. Montero, Corey Lipchik, Melanie Finnigan, Rachel C. Jankowitz, Mohamad A. Salkeni, Sai K. Maley, Shannon L. Puhalla, Fanny Piette, Katie Quinn, Kyle Chang, Rebecca J. Nagy, Carmen J. Allegra, Kelly Vehec, Norman Wolmark, Peter C. Lucas, Ashok Srinivasan, Katherine L. Pogue-Geile

**Affiliations:** 1 NSABP Foundation, Pittsburgh, PA USA; 2 Cleveland Clinic, Weston/Taussig Cancer Institute, Cleveland, OH USA; 3 University Hospitals/Seidman Cancer Center, Case Western Reserve University, Cleveland, OH USA; 4 University of Pittsburgh, Pittsburgh, PA USA; 5 Present Address: University of Pennsylvania Perelman School of Medicine, State College, PA USA; 6 National Institutes of Health, Washington, DC USA; 7 Present Address: Virginia Cancer Specialists, Fairfax, VA USA; 8 UPMC Hillman Cancer Center, Pittsburgh, PA USA; 9 University of Pittsburgh School of Medicine, Pittsburgh, PA USA; 10 International Drug Development Institute, Louvain-la-Neuve, Belgium; 11 Guardant Health, Redwood City, CA USA; 12 University of Florida Health, Gainesville, FL USA; 13 Department of Laboratory Medicine and Pathology, Mayo Clinic, Rochester, MN USA; 14 Autism Impact Fund, Pittsburgh, PA USA

**Keywords:** Metastatic breast cancer, ctDNA HER2 amplification, Clinical trial, Neratinib + t-DM1

## Abstract

**Background:**

We previously reported our phase Ib trial, testing the safety, tolerability, and efficacy of T-DM1 + neratinib in HER2-positive metastatic breast cancer patients. Patients with ERBB2 amplification in ctDNA had deeper and more durable responses. This study extends these observations with in-depth analysis of molecular markers and mechanisms of resistance in additional patients.

**Methods:**

Forty-nine HER2-positive patients (determined locally) who progressed on-treatment with trastuzumab + pertuzumab were enrolled in this phase Ib/II study. Mutations and HER2 amplifications were assessed in ctDNA before (C1D1) and on-treatment (C2D1) with the Guardant360 assay. Archived tissue (TP0) and study entry biopsies (TP1) were assayed for whole transcriptome, HER2 copy number, and mutations, with Ampli-Seq, and centrally for HER2 with CLIA assays. Patient responses were assessed with RECIST v1.1, and Molecular Response with the Guardant360 Response algorithm.

**Results:**

The ORR in phase II was 7/22 (32%), which included all patients who had at least one dose of study therapy. In phase I, the ORR was 12/19 (63%), which included only patients who were considered evaluable, having received their first scan at 6 weeks. Central confirmation of HER2-positivity was found in 83% (30/36) of the TP0 samples. HER2-amplified ctDNA was found at C1D1 in 48% (20/42) of samples. Patients with ctHER2-amp versus non-amplified HER2 ctDNA determined in C1D1 ctDNA had a longer median progression-free survival (PFS): 480 days versus 60 days (*P* = 0.015). Molecular Response scores were significantly associated with both PFS (HR 0.28, 95% CI 0.09–0.90, *P* = 0.033) and best response (*P* = 0.037). All five of the patients with ctHER2-amp at C1D1 who had undetectable ctDNA after study therapy had an objective response. Patients whose ctHER2-amp decreased on-treatment had better outcomes than patients whose ctHER2-amp remained unchanged. HER2 RNA levels show a correlation to HER2 CLIA IHC status and were significantly higher in patients with clinically documented responses compared to patients with progressive disease (*P* = 0.03).

**Conclusions:**

The following biomarkers were associated with better outcomes for patients treated with T-DM1 + neratinib: (1) ctHER2-amp (C1D1) or in TP1; (2) Molecular Response scores; (3) loss of detectable ctDNA; (4) RNA levels of HER2; and (5) on-treatment loss of detectable ctHER2-amp. HER2 transcriptional and IHC/FISH status identify HER2-low cases (IHC 1+ or IHC 2+ and FISH negative) in these heavily anti-HER2 treated patients. Due to the small number of patients and samples in this study, the associations we have shown are for hypothesis generation only and remain to be validated in future studies.

*Clinical Trials registration* NCT02236000

**Supplementary Information:**

The online version contains supplementary material available at 10.1186/s13058-024-01823-8.

## Introduction

In 2013, T-DM1 was the first HER2-targeted antibody–drug conjugate (ADC) granted FDA-approval for late-stage metastatic breast cancer after prior trastuzumab. In 2019, T-DM1 was approved as post-neoadjuvant therapy in patients with residual disease based on the KATHERINE trial, demonstrating that post-neoadjuvant T-DM1 was statistically more beneficial than trastuzumab, preventing recurrence of invasive disease or deaths in patients with residual disease in breast or lymph nodes after treatment with trastuzumab ± pertuzumab (hazard ratio for invasive disease or death, 0.05: 95% CI 0.039–0.64; *P* < 0.001) [1]. KATHERINE required archival HER2-positivity but did not mandate HER2 status at study entry. Because multiple studies have confirmed that HER2 status is plastic with conversion of HER2-positive disease to HER2-low (IHC = 0–1+ or IHC 2+ /FISH-negative) under pressure of therapy [2–4], this raised the question of ADC efficacy in HER2-low patients—either de novo (HR + /HER2-negative) or acquired from conversion of HER2-amplified to HER2-low. There are now several breast cancer-targeting ADCs in the pipeline in addition to the newly approved trastuzumab deruxtecan (T-DXd). Initial approval of T-DXd was for metastatic HER2-positive breast cancer after prior progression on multiple lines of anti-HER2 therapy (DESTINY-Breast01) [5]. In DESTINY-Breast03, T-DXd improved PFS and OS compared to T-DM1 [6] in patients with metastatic disease with progression on trastuzumab. In heavily pretreated HER2-low breast cancer patients, T-DXd was evaluated in a single arm phase II study. The objective response rate (ORR) to T-DXd by central review was 37%, with median duration of response of 10.4 months [7]. DESTINY-Breast04, a randomized, multicenter trial in patients with unresectable or metastatic HER2-low breast cancer, reported highly significant improvements in PFS and OS in patients receiving T-DXd compared to physician choice of treatment [8]—a particularly striking observation, because neither trastuzumab nor T-DM1 has shown consistent activity in HER2-low populations [9]. HER2 status (expression, mutation, amplification) is thus emerging as a predictor of clinical efficacy for anti-HER2-therapy. In our NSABP phase Ib trial of HER2-targeted therapies using T-DM1 + neratinib in HER2-positive patients, we showed a discordance in HER2 status between archival tissue and a liquid biopsy obtained at study entry. Loss of ctHER2-amp occurred in 63% (17 of 27) patients. Deeper and more durable responses were observed with T-DM1 + neratinib in patients with ctHER2-amplification [10]. We now report on an expanded cohort. Our aims were to: (1) confirm activity of T-DM1 + neratinib in patients progressing on a taxane with trastuzumab + pertuzumab (HP), (2) evaluate discordance in HER2 amplification between archived tissue, contemporaneous tissue, and blood, and (3) compare mutation and gene-expression profiles at different time points. Finally, in a subset of patients, we assessed response by RECIST 1.1 with the Guardant Molecular Response score [11, 12].

## Methods

### Trial design

FB-10 was a single-arm, nonrandomized, unblinded clinical trial approved by participating institutions’ institutional review boards. Written informed consent was required. FB-10 was conducted according to Good Clinical Practices and the Declaration of Helsinki.

**Fig. 1:**
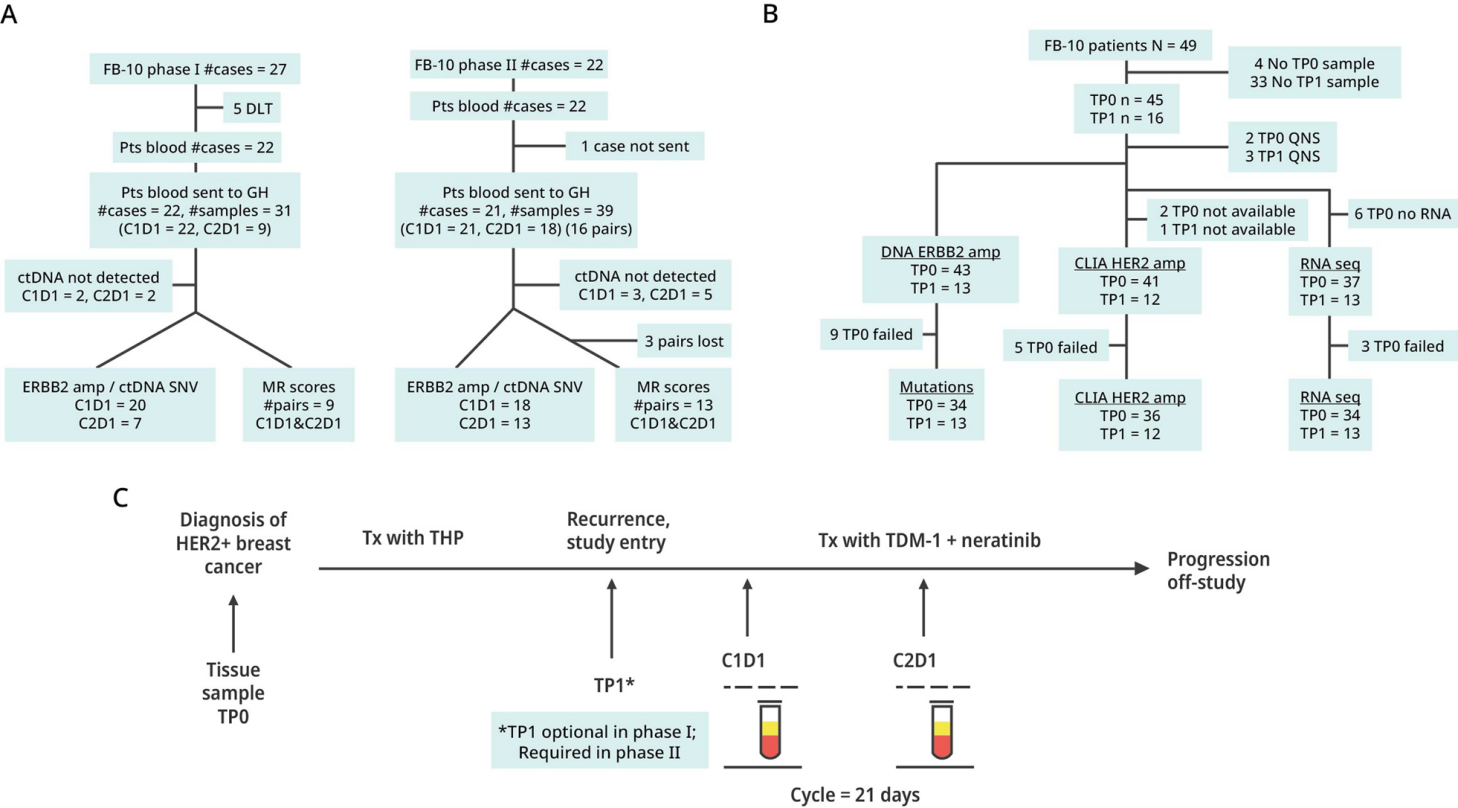
Remark Diagram of Blood and Tissue Samples: NSABP FB-10. **A** Blood samples collected from patients enrolled into FB-10 phase Ib and phase II, and successful assays for ctDNA analysis of HER2 amplification with Guardant360 assays. **B** Tissue samples collected from patients enrolled into FB-10 and their samples that were profiled for mutations and whole transcriptomic analysis and for ERBB2 amplification status with CLIA and AmpliSeq assays. **C** The timing and type of sample collections (tissue: TP0 or TP1, or blood: C1D1 or C2D1) are shown

The phase Ib trial was a dose escalation study evaluating T-DM1 + neratinib in women with metastatic HER2-postive breast cancer based on local determination of HER2. Patients received 3.6 mg/kg T-DM1 intravenously on a 3-week cycle and oral neratinib was taken daily in one of four dose cohorts (120, 160, 200 and 240 mg). Twenty-seven patients enrolled, with 5 experiencing a dose-limited toxicity. Three withdrew early for other reasons (Fig. 1). Nineteen patients were evaluable for response, which required follow-up imaging after the second cycle of treatment (6 weeks). The recommended phase II dose of neratinib was determined to be 160 mg/d [10]; however, we did not detect a dose response, i.e., neratinib at 120 mg/d was as effective as higher doses and less toxic. [10]

The phase II expansion included all patients (N = 22) who received at least one dose of study therapy in the analysis of safety and efficacy. Eligibility criteria were identical in phase Ib and phase II [10]. All eligible patients had prior HP and a taxane as neoadjuvant therapy or for de novo metastatic disease, had measurable disease, were ECOG PS ≤ 2, with adequate hematologic, renal, and liver function. Patients with known stable brain metastases were eligible. Brain imaging at entry was not required. Treatment in phase II included T-DM1 at 3.6 mg/kg iv q 3 weeks and neratinib at 160 mg/day. Primary diarrhea prophylaxis was mandated as described in phase I [10].

### Safety assessment

Safety assessment was similar in phase Ib and phase II including physical examination, interim history, and laboratory assessments. Patients remained on treatment until progressive disease or discontinuation because of withdrawal, physician discretion or toxicity. For phase Ib patients, adverse event (AE) assessment occurred on days 1, 8, and 15 of cycle 1; on day 1 of each cycle and for 30 days after therapy discontinuation or when alternate therapy began. Phase II AE assessments were made on day 1 of each cycle.

### Response evaluation

In phase Ib, patients were assessed for best response beginning with their first follow-up scan after 2 cycles (6 weeks). Response by RECIST v1.1 was complete response (CR), partial response (PR), stable disease (SD), or progression (PD). In phase II, imaging studies were performed after every 3 cycles (9 weeks). The clinical benefit rate included all CR, PR, and SD patients with duration ≥ 180 days. Patients with stable disease of < 180 days were included with progressive disease patients. A confirmatory scan at least one month after the best response was not required in this study, perhaps accounting for partial responses of short duration in several patients.

### Blood and tissue collection

Blood samples were required before treatment at cycle 1, day 1 (C1D1), and after treatment at cycle 2 day 1 (C2D1) for all patients in phase Ib and II (Fig. 1A). Archived tissue (TP0) of diagnostic blocks or slides were required on all phase Ib and II patients which included 27 patients from phase Ib and 22 patients in phase II. (Fig. 1B). Contemporaneous biopsy specimens or slides at study entry (TP1) were optional in phase Ib but in phase II, after enrollment of the first 6 patients, the study was amended to require a study entry biopsy. The timing and type of collections of samples are shown in Fig. 1C.

### ctDNA assessment

Samples were analyzed by the Guardant360 assay (Fig. 1A), which detects single-nucleotide variants, indels, fusions, and copy number alterations in 74 genes. For HER2 amplification, a cutoff of ≥ 2.14 was used. Where amplification could not be determined because of failed assays or no blood, samples were categorized as indeterminant. Guardant Health (Guardant360 assay) is Clinical Laboratory Improvement Amendments (CLIA)-certified, College of American Pathologists-accredited, New York State Department of Health-approved laboratory.

### ctDNA molecular response

Guardant360 Molecular Response is a next generation sequencing (NGS)‐based liquid biopsy that assesses changes in tumor‐derived cell‐free DNA (ctDNA) between baseline and an early on‐treatment timepoint in patients with solid tumor malignancies. It employs an algorithm to identify informative somatic single nucleotide variants (SNVs), insertions/deletions and gene fusions and calculates the percent ctDNA change between the two timepoints based on the mean variant allele frequency (VAF) between two samples (mean VAF_2_/mean VAF_1_) -1 × 100%. Using the Molecular Response panel and the Guardant bioinformatics pipeline, the change in ctDNA levels between baseline and the initial follow-up scan (6 weeks in phase I and 9 weeks in phase II) was calculated and the change in VAF determined. Molecular Response is calculated as the ratio of mean VAF on-treatment to baseline with a cutoff of 50%. Decreases in ctDNA of 50%‐100% during this timeframe are associated with clinical benefit in patients on anti‐cancer therapies [11, 13, 14]. Kaplan–Meier curves for PFS are generated for patients above and below a Molecular Response cut off. [11]

### Isolation of nucleic acid

Tumor regions, defined by a certified pathologist, were macrodissected. DNA and RNA were isolated using the Qiagen AllPrep DNA/RNA kit, following the manufacturer’s recommendations but eliminating the xylene wash in the first step. Separate tissue sections were used for RNA and DNA isolation.

### Whole transcriptomic profiling

10–30 ng of RNA from the phase II samples was reverse transcribed. cDNA libraries were constructed using whole transcriptomic Ampli-Seq kits, following the manufacturer’s instructions without a fragmentation step due to the small size of the RNAs. This same method did not work well for the phase Ib RNAs. To overcome this problem, phase Ib RNAs were made library-ready via the HTG EdgeSeq system and the HTP panel, which includes probes to interrogate 19,398 genes representing most of the human transcriptome (details in Additional file 1).

Breast cancer molecular subtypes were determined by applying the AIMs signature [15]. The 8-gene trastuzumab-benefit groups were determined using a validated signature. [16, 17]

### HER2 amplification status and analysis of variants in tissues

DNA sequencing was performed using a custom Ampli-Seq panel referred to as the NAR panel, amplifying 3,847 amplicons with 94.25% coverage of exons from 117 genes in HER2-activated pathways (Additional file 1: Table S1). The panel was designed using the Thermo Fisher Ion AmpliSeq™ Designer tool (https://www.ampliseq.com). Libraries were constructed using 10 ng of DNA using the Ion AmpliSeq™ kit for Chef DL8. The Ion Chef instrument was used to template and load samples on Ion 550 chips. Up to 32 samples were barcoded, pooled, and sequenced on the S5 sequencer (ThermoFisher) following manufacturer’s instructions.

We have used two different criteria to identify variants in FB-10 tissue. For a conservative approach to variant selection, we selected variants with VAF ≥ 10% and for a less restrictive option we selected variants with VAF ≥ 5%. Additional details are included in Additional file 1 and the rationale for these approaches is discussed.

Ion Torrent data and the Ion Reporter software were used to determine HER2 copy number.

### HER2 IHC FISH

HER2 status was also assessed in tissue samples with IHC and reflexively for FISH at the discretion of the Director at the CLIA laboratory (Magee Women’s Hospital, University of Pittsburgh Medical Center). Nine samples were equivocal IHC 0 or 1+ due to poor tissue quality prompting examination with FISH. Based on FISH, four were included as HER2-positive.

### Statistical analysis

Phase Ib safety, tolerability, efficacy, and recommended phase 2 dose (RP2D) of neratinib in combination with T-DM1 was previously reported [10]. In the phase II expansion cohort, in which neratinib was administered at the RP2D of 160 mg/d, the intention was to confirm clinical efficacy and tolerability of the combination and to extend the correlative findings. Given the small sample size, the endpoint analyses remain descriptive.

The aim of the single-arm phase II expansion was to rule out the null hypothesis that the ORR was 25% with the alternative hypothesis of an ORR of 45%. With these assumptions, the sample size required was 22 and the decision rules are to declare success if > 8 responses; to declare failure if < 7 responses; and to consider the trial inconclusive if 7 or 8 responses (7/22 = 32%, 8/22 = 36%). At the outset of the study, we did not anticipate the large number of patients with loss of HER2-amplification in blood as determined by the Guardant assay. Thus, the subset analyses based upon HER-amplification detected in blood were performed post-hoc.

## Results

### Patient characteristics

In the phase Ib portion of this study, 27 patients were enrolled between February 2015 and July 2017. Nineteen patients were evaluable having had at least one follow-up imaging study; three patients withdrew from the study in cycle 1 and 5 patients with a dose-limited toxicity in cycle 1 did not have an imaging assessment. All phase II patients who received at least one dose of study drugs were included in the analysis. Twenty-two patients were evaluable for toxicity and 20 were evaluated for efficacy with at least one scan performed after their third cycle. Two non-evaluable patients who withdrew from the study did not have their first scan but are included as PD. Median age was 55.5 years (range 32–70). Hormone status (ER and/or PR) was positive in 13 patients and negative in 9. All patients were HER2-positive at baseline by local determination (Additional file 1: Table S3).

### Safety assessment

Similar to phase Ib patients, diarrhea was the most frequent toxicity in phase II: grade 2, 6 patients (27%); grade 3, 8 (36%). Other grade 3/4 toxicities included: thrombocytopenia, 2 patients (10%); transaminase elevation, 3 patients (15%); and pneumonitis, 1 patient (5%). There were no unanticipated toxicities in the phase II expansion.

### Efficacy

Among 19 evaluable patients in phase Ib, there were 3 CRs and 9 PRs for an ORR of 63% (12/19) [10]. In phase II, including all patients who received at least one dose of therapy, there were 2 CRs, 5 PRs for an ORR of 32% (7/22), and 3 SDs of 180 days or longer making the clinical benefit rate (CBR) 45% (10/22). In phase Ib and II, nine patients had sustained objective responses lasting approximately 1 year or longer (range 343–1453 + days, Additional file 1: Table S4; Additional file 2: Table S5). Treatment was discontinued at or before the first scan in 15 patients for a variety of reasons, including 5 DLTs (all in phase I) and 10 with clinical progression in phase I and II.

### ctDNA clearance and treatment response

Because clearance of ctDNA has been associated with treatment response, we compared the outcomes of patients who were positive or negative for ctDNA after study treatment. The response rate among the ctDNA-positive patients who were still ctDNA-positive after study therapy was 9/19 (47%), but the ctHER2 DNA-positive patients who became ctDNA-undetectable at C2D1, the response rate was 6/6 (100%), demonstrating that the loss of ctDNA was associated with a very good response.

### HER2 amplification in tissues and blood

**Fig. 2:**
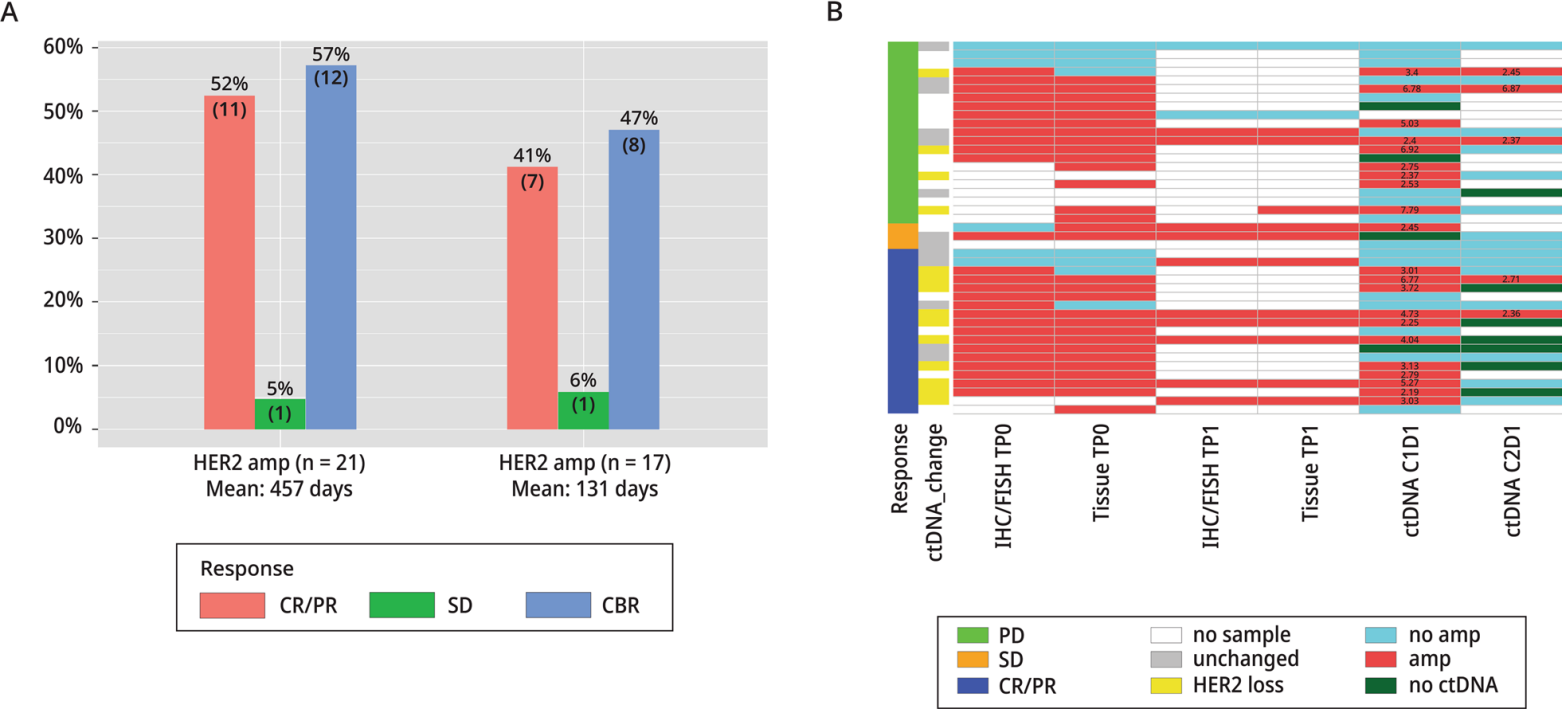
Amplification Status of Tissues and Blood: NSABP FB-10. **A** Response rates (CR/PR, CBR, and SD) for FB-10 HER2-amplified and non-amplified patients based on ctDNA results. **B** HER2-amplification status of FB-10 tumor tissues based on CLIA tests (IHC/FISH) and Ampli-Seq (Tissue) in baseline (TP0) and study entry (TP1) samples are shown. HER2-amplification status was determined in ctDNA at C1D1 and at C2D1 with the Guardant360 assays. Responses, amplification status, and changes in copy number in ctDNA between C1D1 and C2D1 are indicated as shown in the legend

We assessed the HER2 amplification status of TP0 and TP1 with a CLIA HER2 IHC/FISH assay, and with an Ampli-Seq NGS assay. These tissue samples were also compared to the HER2 amplification status in blood samples collected at C1D1 and C2D1 (Fig. 2B). There was good concordance between the CLIA HER2/FISH and Ampli-Seq assays (100% in TP1 tissues and 85% in all tissues), which demonstrated the technical accuracy of the Ampli-Seq. Concordance between TP0 tissue with IHC/FISH and C1D1 ctDNA was 71% (20/28). Concordance between C1D1 and C2D1 was 54% (14/26). Anti-HER2 treatment is potentially the causal reason for the discordance between C1D1 and C2D1, which showed a total loss of ctDNA in some samples and a loss of HER2 amplification in others.

Using the Guardant360 assay cut point of 2.14 for amplification among 43 C1D1 samples (22 in phase Ib, 21 in phase II), 6 patient samples were indeterminate (including 4 for which somatic mutations were not detected) (Fig. 2B**,** dark green), 1 was not evaluable (NE), and 1 other failed quality control. Among the remaining 37 samples, there were 21/37 (57%) patients with amplification and 17/37 (46%) without. The objective response (CR, PR) rate was 55% (11/20) in amplified patients and 41% (7/17) in non-amplified patients. The CBR in patients with ctHER2-amplification was 12/21 (57%) and in non-amplified patients it was 8/17 (47%). There was one patient with SD who was ctHER2-amp indeterminate. Mean duration of response (CBR) was substantially longer in amplified patients, 457 days compared to 131 days in non-amplified patients (*P* = 0.008) (Fig. 2A; Additional file 1: Table S4).

**Fig. 3:**
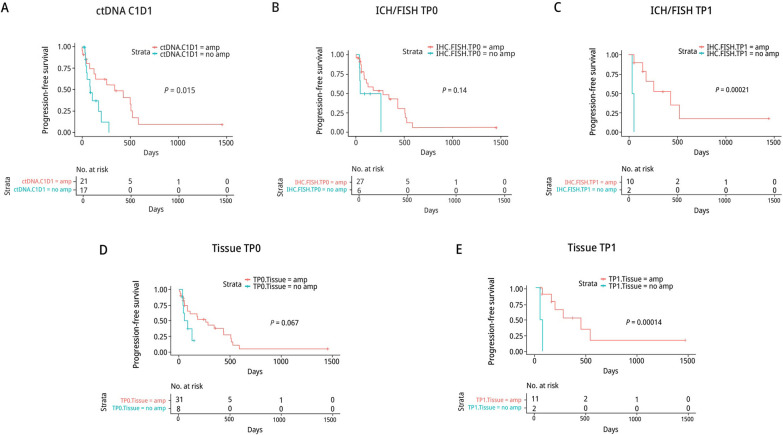
Association of HER2-amplification Status with Patient Outcomes: NSABP FB-10. **A** Kaplan-Meier plots of patients with or without HER2 amplification in ctDNA or in TP0 tissue (**B** & **D**), or in TP1 tissue (**C** & **E**) based on IHC/FISH (**B** & **C**) and on Ampli-Seq (**D** & **E**)

We compared progression-free survival (PFS) of patients whose ctDNA or tumor tissues had HER2 amplification to patients with no HER2 amplification. Patients with ctHER2-amp at C1D1 or in their TP1 tumor tissue had a significantly longer PFS than patients with no HER2 amplification **(**Fig. 3A–E**)**.

In phase I and II there were 26 C1D1 and C2D1 pairs, 15 with and 11 without ctHER2-amp at C1D1. Among the 15 with ctHER2-amp at C1D1, 14 showed HER2 loss at C2D1 as defined by a loss ≥ 28% of HER2 copy number or no ctDNA detected. The ORR among these 14 patients was 71% (10/14). Of the 10 responders, 5 cleared ctDNA completely by C2D1, 3 had detectable ctDNA but no ctHER2-amp, and 2 were HER2-positive but the amplification level in C2D1 had decreased dramatically (Fig. 2B; Additional file 2: Table S5). The 2 remaining patients with detectable ctHER2-amp with no loss of HER2 amplification had PD, suggesting that a loss of ctDNA and/or a loss of HER2 ctDNA amplification was a marker for a good response to study therapy. However, in 11 patients with no HER2 ctDNA amplification at C1D1, the ORR was 45% (5/11), indicating that some non-amplified tumors were responsive to study treatment.

### Molecular response by ctDNA

**Fig. 4:**
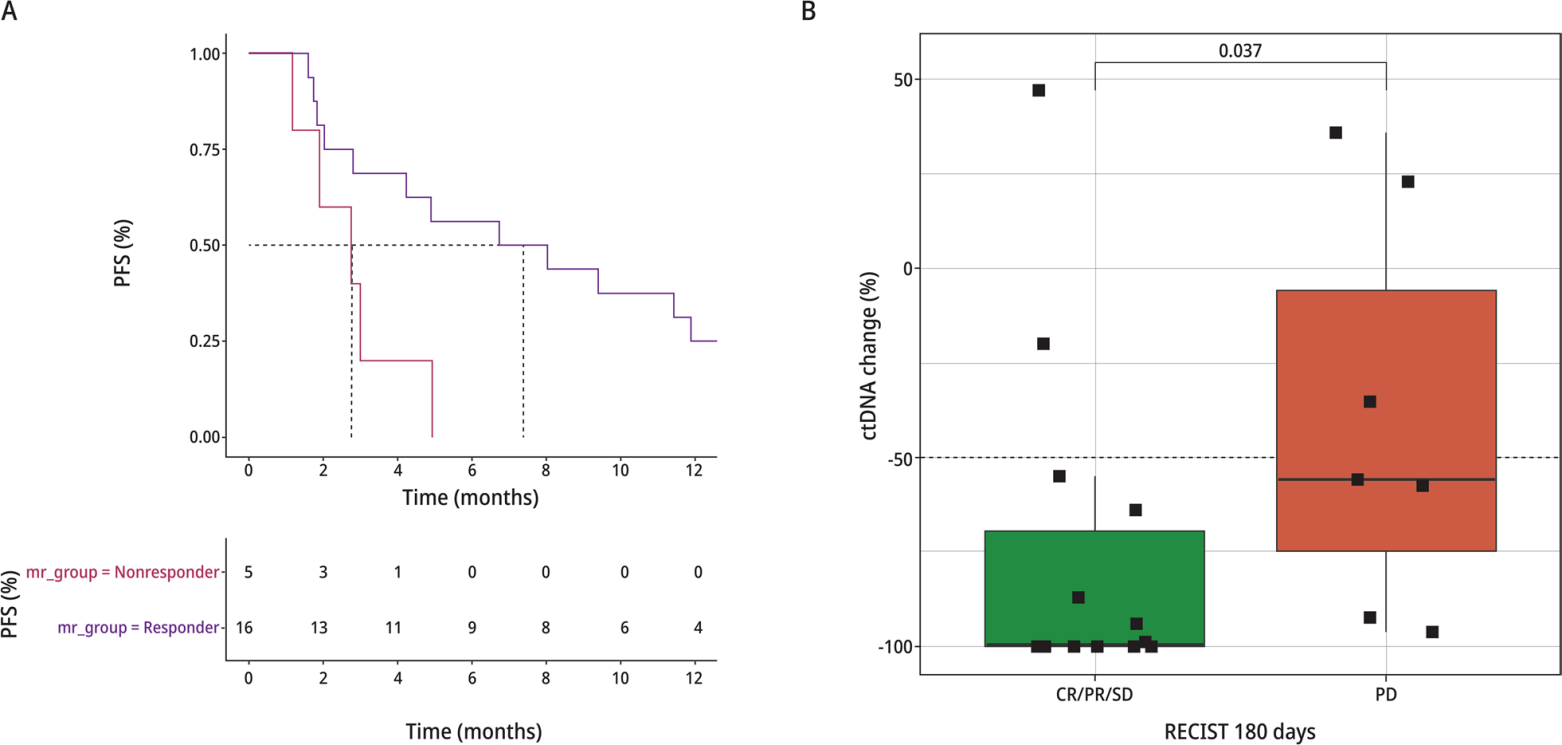
Molecular Response and Patient Outcomes: NSABP FB-10.** A** Kaplan–Meier curves showing association of MR with PFS using a molecular response cutoff of 50%. **B** Association between molecular response and best RECIST response

We assessed the association between molecular response and objective radiologic response (Fig. 4A). A total of 21 patients (9 phase Ib and 12 phase II) had paired samples that met criteria for assessment of molecular response. Criteria included ≥ 1 alteration present in one of the paired samples plus a mutant molecule count of ≥ 15 in either sample. Molecular responders demonstrated a longer PFS compared to non-responders (median PFS 7.4 vs. 2.8, HR 0.28, 95%CI 0.09-0.90, P=0.033 using Wilcox test). We also examined the association between molecular response and best RECIST response. Patients with CR/PR/SD had significantly lower Molecular Response values compared to patients with PD (*P* = 0.037; Fig. 4B**).**

### Mutations/variants in tissues and ctDNA

Because ERBB2 is the target of the study therapies, we have examined both tissue and ctDNA for mutations in the ERBB2 gene. No ERBB2 variants in tissue at a VAF of ≥ 10% were observed, however, in ctDNA 3 nonsynonymous, ERBB2 variants (I767M, V777L, and S310Y) were detected in the C1D1 samples from 3 patients. These variants have been associated with sensitivity to neratinib in breast cancer patients [18]. In this study, the patients whose tumors had a V777L or a S310Y mutation had a PR, but the one patient with a I767M mutation had PD with brain metastasis. The tumor with the I767M mutation also had a P1233L mutation [19]. Interestingly, in an exhaustive meta-analysis of 37,218 patients, including 11,906 primary tumor samples, 5,541 extracerebral metastasis samples, and with 1485 brain metastasis samples found that a nearby ERBB2 mutation (P1227S) was the only mutation restricted to brain metastasis. It is unknown whether any of these mutations played a role in the patient responses or the course of disease, but it is of interest to note them [20].

**Fig. 5:**
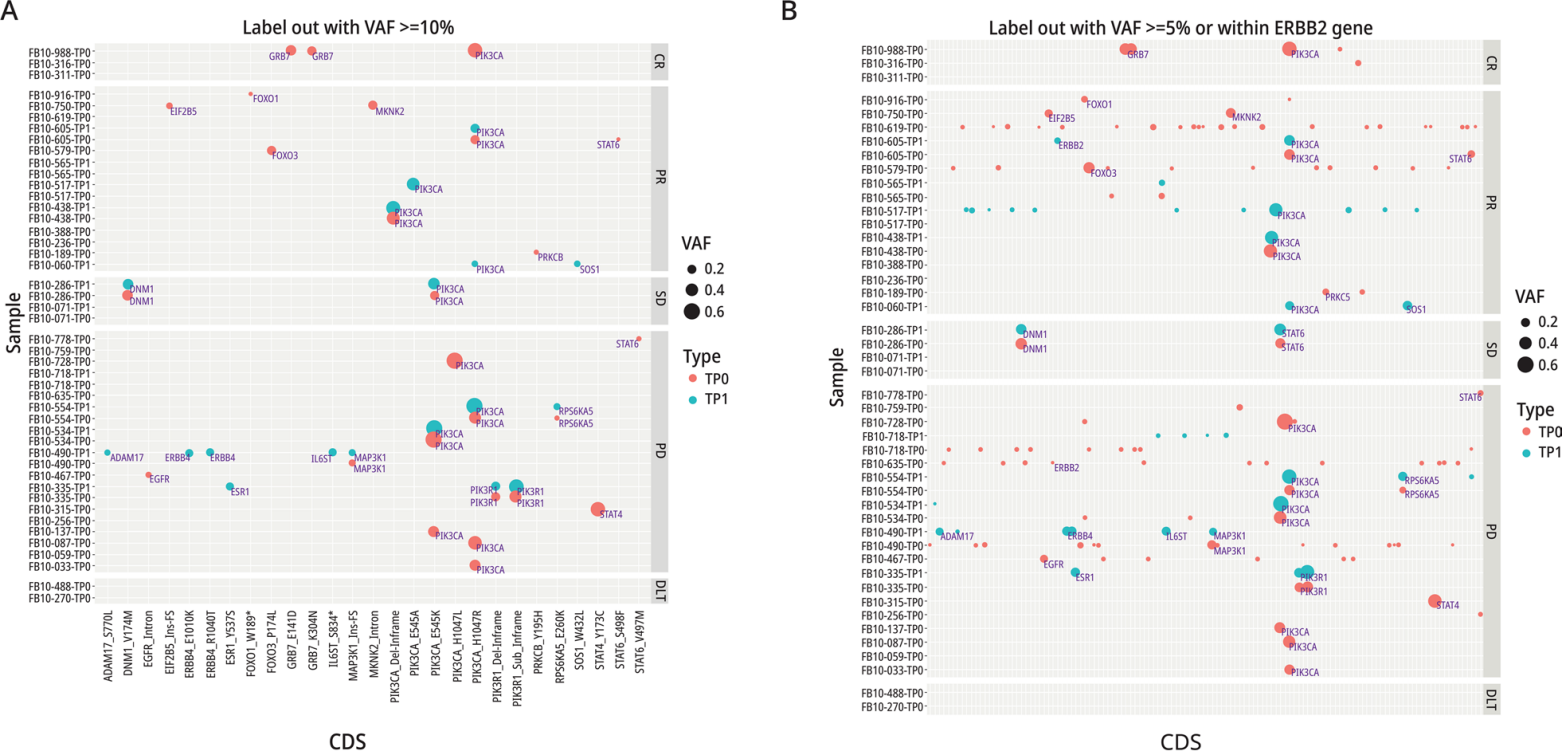
Variant Alleles in Patients and their Responses: NSABP FB-10. **A** Variants detected with a VAF of ≥ 10% in patients with PD, SD, PR or CR. *indicates a stop codon. **B** Variant alleles with a VAF of ≥ 5% in patients with PD, SD, PR or CR

We examined DNA variants in all available TP0 and TP1 tissues using our NAR Ampli-Seq panel, which included ESR1, HER2, and 115 other genes in HER2-activated pathways. Based on our stringent criteria for variant detection, i.e., VAF ≥ 10%, plus other criteria as described in Additional file 1, we identified 27 variants among 28 samples, representing 21 patients **(**Fig. 5A**)**.

The frequency of PIK3CA mutations among all sequenced patients was 34% (12/35), similar to that seen in other studies of unselected metastatic and early-stage breast cancer patients (cBioPortal). All of the mutations were in exons 9 and 20 at amino acid 545 and 1,047, respectively. These PIK3CA variants also have the highest VAFs (ranging from 10 to 72% across samples), however, PIK3CA mutations do not appear to influence patient outcomes, because response rates between PIK3CA mutant and WT tumors were similar: 42% (4/12) versus 45% (14/31), respectively. In one case, a PIK3CA mutation was detected only in TP1 but this patient had a PR, again indicating that PIK3CA is not a resistance marker for study therapy. Variants detected only in PD or CR patients represent potential resistance or sensitivity markers, respectively, to study therapy. Mutations detected only in TP1 samples among the 12 paired TP0/TP1 cases, included ADAM17_S770L, ERBB4_E1010K, ERBB4_R1040T, and IL6ST_S834* in one sample and an ESR1_EY537S mutation in another (Fig. 5A). Both patients had PD, perhaps indicating that these mutations may have emerged in response to prior therapies. Details of variants are presented in Additional file 1.

### Whole transcriptomic profiling

We examined the PAM50 subtypes and the 8-gene trastuzumab benefit signature in all available tissues [16]. Among the 29 patients with response and gene expression data for TP0 tissue, we found that 19/34 (56%) were HER2E, 8 (24%) were luminal B, 4 (12%) were basal, 2 (5.9%) were normal, and 1 (2.9%) was luminal A. Patients with luminal subtype tumors had a lower CR/PR response rate (1/8 [12.5%]) than patients with a non-luminal subtype (12/23 [52%]) (Additional file 2: Table S5). Among the TP1 samples with gene expression data, the frequency of CR/PR was 1/4 in luminal patients and 5/9 in the non-luminal patients. Intrinsic subtypes differed between TP0 and TP1 tissues in some cases (Additional file 1: Table S4). Although numbers are small, the frequency of CR/PR rates were consistently lower among the luminal patients than non-luminal patients.

The 8-gene trastuzumab signature is a validated signature for identifying patients with large-, moderate- or no-benefit from trastuzumab when added to chemotherapy in the adjuvant setting [16, 17] and has been shown to associate with pCR rates in the neoadjuvant setting [21, 22]. We questioned whether this signature may also show an association with response in the metastatic setting. Among the large-, moderate- and no- benefit groups the percent of CR/PR patients was 67% (4/6), 50% (9/18), and 29% (2/7), respectively (data in Additional file 2: Table S5).

**Fig. 6:**
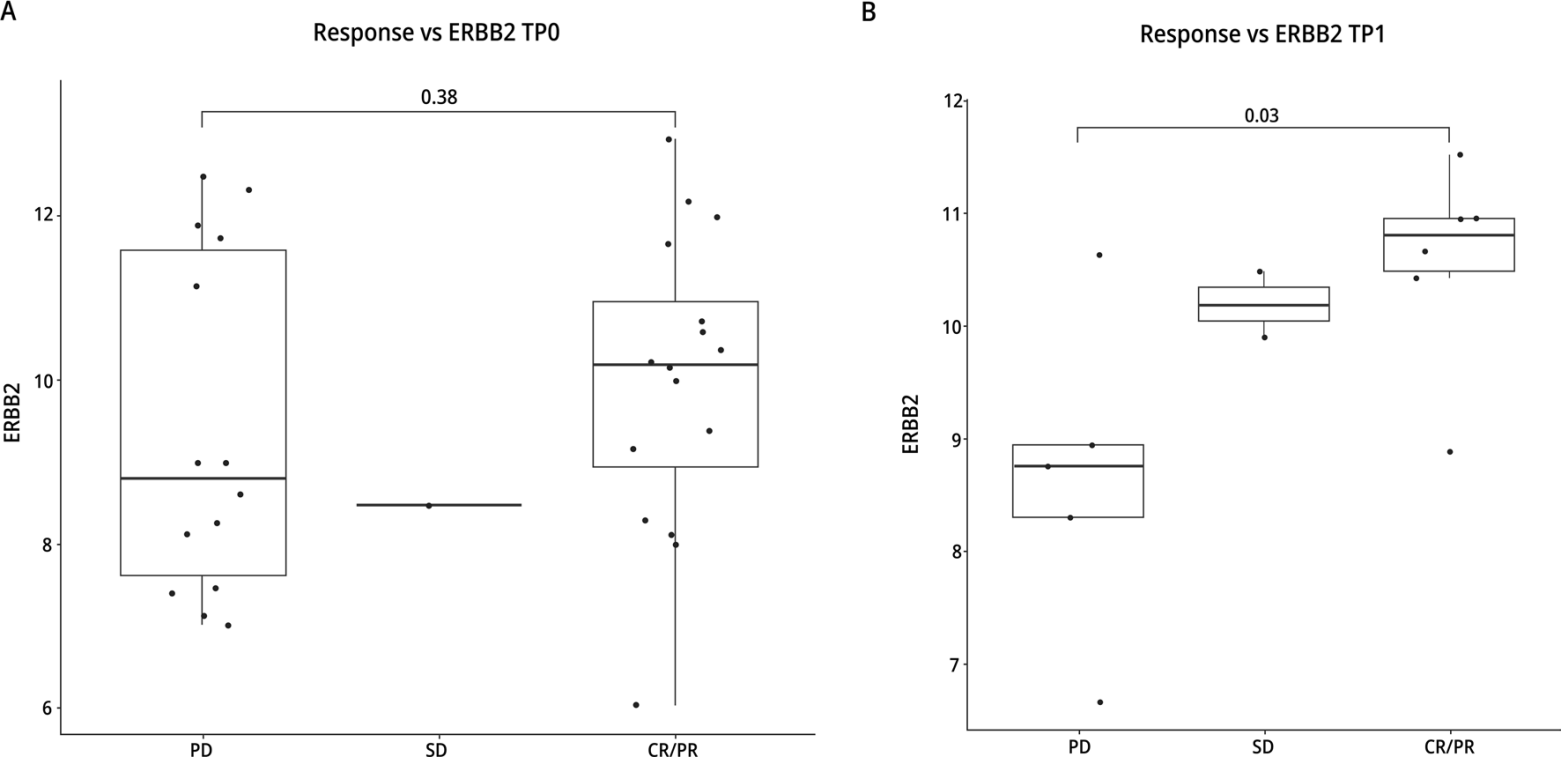
RNA Expression Levels and Response to Therapy. RNA expression levels in TP0 tissues (**A**) and in TP1 tissues (**B**) from patients with PD, SD or CR/PR. The units for RNA expression were log 2 expression values

As expected, the level of HER2 RNA increased as the IHC status increased (i.e., 0, 1 + , 2 + , to 3 +) (Additional file 1: Fig. S2). In TP1 samples they were concordant with patient responses suggesting that HER2 RNA expression in study entry is associated with response to T-DM1 + neratinib **(**Fig. 6**).**

Significant differences were detected in RNA levels between IHC 1 + and 3 + and between 2 + and 3 + but not between 0 and 1 + nor between 1 + and 2 + (Additional file 1: Fig. S3). Although numbers are limited, these data show that the RNA levels are not different between 0 and 1 + . These patients may benefit from treatment with other ADCs. The DAISY and DESTINY-Breast04 trials signal that T-DXd may have significant activity in HER2-low patients [8, 23].

## Discussion

Approximately 35% of HER2-positive patients may have a loss of HER2 amplification after undergoing chemotherapy + anti-HER2 therapy [2, 3]. In a retrospective analysis of 525 patients who received neoadjuvant chemotherapy (NAC) + HP, 141 patients with residual disease had HER2 status determined pre-and post-NAC-HP. HER2 was concordant (positive/positive) in 84/141 (60%). HER2 protein expression was lost (IHC 0) in 13/57 (23%) and designated as HER2-low in 44/57 (77%) including IHC 1 + in 31 and IHC 2 + /FISH non-amplified in 13 [4]. HER2 intratumoral heterogeneity is likely one cause of discordant HER2 status between primary and post-treatment residual or metastatic disease [24]. Other possibilities include decreased HER2 expression, which could be a transient change or a result of the selection of HER2-low subclones [4].

We have assessed HER2 status before and after pre- and post-study therapy in not only solid tissue but also blood. We have determined the HER2 status in tissues with CLIA IHC/FISH assays, which is the gold standard for HER2 assessment, plus with Ampli-Seq, because it provided a quantitative analysis of HER2 copy number with a greater dynamic range. Ampli-Seq was able to detect a decrease in HER2 copy number in samples that had not lost HER2 amplification based on IHC/FISH. We have also monitored HER2 status in liquid biopsies, which has several advantages over genomic analysis of tissues. Blood has exposure to all potential metastatic sites allowing for the detection of different variants from different metastatic sites. Thus, blood may be more representative of the metastatic tumor than examination of a single biopsied lesion, and may reflect tumor evolution and intratumoral heterogeneity [25]. Blood samples are more easily collected, making multiple serial collections possible. Collecting multiple serial tissue samples is impractical, costly, and represents a much greater risk to patients than does serial collection of blood. The assessment of ctDNA is a powerful tool, showing very promising results to monitor tumor recurrence and response to therapy, but it does not yet replace the current gold standard, IHC/FISH, for the assessment of HER2 status in solid tumors. However, the monitoring of the HER2 status in ctDNA does provide an indicator of tumor response to therapy.

In our phase Ib/II study, HER2 tissue was amplified in the baseline samples (TP0) (pre- anti-HER2 therapy) in all patients by local determination, however, in liquid biopsies at C1D1 after chemotherapy + HP, HER2-amplification was detected in only 20/42 (48%) of patients. Patients with ctHER2-amp versus non-amplified HER2 ctDNA determined in C1D1 ctDNA had a longer median PFS, 480 days versus 60 days (*P* = 0.015). It is expected that patients with HER2 amplification would respond to study therapy (chemotherapy + HP).

Loss of HER2 amplification observed after one cycle of study therapy may indicate that responders are either clearing ctDNA completely or that the amplification falls below the limit of detection. In the 5 cases who were ctHER2 DNA amplified, and completely cleared ctDNA, the response rate was 100%.

We applied a Molecular Response VAF ratio calculation to measure the change in ctDNA from baseline to C2D1, with the hypothesis that an early decrease in ctDNA levels would predict response to T-DM1 + neratinib therapy, as measured by PFS and RECIST response. Indeed, Molecular Response was associated with both PFS and best response to therapy. This should be confirmed in larger dataset, however, our findings are in line with other studies demonstrating the ability of ctDNA to predict short- and long-term efficacy. Early data from the PADA-1 trial suggests that changing therapy based on alterations detected in ctDNA, prior to evidence of progression via imaging, may provide clinical benefit. In that trial, patients with ER + /HER2-negative metastatic breast cancer being treated in the first line setting with an aromatase inhibitor (AI) + palbociclib were monitored for hotspot ESR1 alterations via ctDNA using digital droplet PCR (ddPCR). Patients with rising ESR1 VAF on therapy, but no synchronous evidence of disease progression via RECIST 1.1, were randomized to either continue receiving an AI + palbociclib or switched to fulvestrant + palbociclib. PADA-1 met its primary efficacy objective, with patients randomized to receive fulvestrant + palbociclib having a significantly longer PFS compared to those who stayed on an AI + palbociclib (median PFS 11.9 months [95% CI 9.1–13.6 months] versus 5.7 months [95% CI 3.9–7.5 months]; stratified HR 0.61 [95% CI 0.43–0.86], two-sided *P* = 0.004) [26]. More data on mutational evolution is needed to determine whether similar strategies employing ctDNA to inform change in therapy will be broadly applicable across breast cancer subtypes and therapy classes in order to further prolong OS.

Although a cross comparison of studies can be problematic, phase II and III studies suggest that as patients are more heavily treated with anti-HER2 regimens, the PFS and ORR decrease with each subsequent anti-HER2 therapy [27–30]. However, in a phase III randomized trial of trastuzumab deruxtecan (T-DXd) versus trastuzumab emtansine (T-DM1) in patients (N = 524) whose disease progressed on anti-HER2 therapy, the reported ORR for patients treated with T-DXd or T-DM1 were 79.7% versus 34.2%, respectively. The landmark analysis of PFS at 12 months was 75.8% with T-DXd as compared to 34.1% with T-DM1 (HR 0.28, 95% CI 0.22–0.37; *P* < 0.001) [6]. Although both T-DXd and T-DM1 have a trastuzumab backbone, there are substantial differences in the linker-payload chemistry, which favors an increased intracellular payload and a bystander effect with T-DXd [31, 32].

We have shown in our study that the benefit from T-DM1 + neratinib is limited in HER2-non-amplified tumors. This finding is consistent with a study reporting a PFS with T-DM1 of 1.5 months [33] in patients discordant for HER2 in primary and metastatic tissue (HER2-positive/negative). Although loss of HER2-amplification appears to be one mechanism of resistance to T-DM1, half of the patients with HER2-amplified tumors did not respond to T-DM1 + neratinib, indicating that resistance to T-DM1 is not limited to loss of HER2-amplification. Many other mechanisms of resistance to T-DM1 have been proposed, such as altered cellular uptake, intracellular transport, and metabolism of the payload. [32]

Based on the low level of activity of T-DM1 monotherapy in patients failing HP, we speculate that the combination of T-DM1 and neratinib is more effective than T-DM1 monotherapy. We are unaware of any trials in HER2-positive breast cancer in which patients progressing on HP have been randomized to compare the response to T-DM1 as monotherapy with a combination of T-DM1 and an irreversible tyrosine kinase inhibitor (TKI). However, in preclinical lung models with ERBB2 mutation and/or amplification, the combination of T-DM1 + neratinib did show increased efficacy over monotherapy. Anecdotally, enhanced efficacy was demonstrated in a breast cancer patient progressing on monotherapy with T-DM1 who then responded with addition of neratinib. The mechanism of action of the antibody–drug conjugates (ADC) such as T-DM1 and T-DXd involves the recognition and binding of the trastuzumab backbone to the extracellular HER2 surface receptor. The ADC-protein complex is internalized with cleavage of the cytotoxic payload. Irreversible TKIs such as neratinib and afatinib (unlike reversible TKIs such as lapatinib) have been shown to enhance HER2 internalization and lysosomal sorting, which has the potential to increase uptake of bound ADC and release of their cytotoxic payload [34].

The KATHERINE [6] study established T-DM1 as a standard of care in early HER2-positive breast cancer patients with residual disease after neoadjuvant therapy [1]. DESTINY-Breast03 has clearly shown superiority of T-DXd over T-DM1 in HER2-positive metastatic disease. The currently recruiting DESTINY-Breast05 study will compare T-DXd with T-DM1 in high-risk HER2-positive patients with residual disease following NAC-HP (NCT04622319). Another study, DESTINY-Breast06 (NCT04494425), will address the question of HER2-low (IHC 1 + or IHC 2 + /FISH-negative or HER2 IHC > 0, < 1 +) in patients with metastatic hormone-positive disease with progression on at least two lines of endocrine therapy comparing T-DXd with investigator’s choice of chemotherapy [31]. The 30-40% of HER2-positive patients with residual or metastatic disease after neoadjuvant therapy who have lost HER2 amplification, while not directly being addressed with these ADCs studies, warrant further investigation with newer generation ADCs.

Our study has several limitations including the non-randomized design, the logistic difficulties in obtaining samples of blood and tissue on all patients and the small sample size, which limited its power and the ability to perform multivariant analysis. However, the strengths of our study include the multiple temporal sample collections, multiple assessments of HER2 status, and molecular assessment of DNA in both tissue and blood.

Despite the limitations of our study, the findings have generated several hypotheses that should be further investigated. First, retrospective confirmation in a large phase III study that loss of HER2-expression under the pressure of therapy can be detected with liquid biopsy; second, the overall response, depth, and duration of response to anti-HER2 therapy is greater in patients with HER2-amplified than in non-amplified patients; third, the activity of T-DM1 or other ADCs with a trastuzumab-backbone may be enhanced with addition of an irreversible TKI such as neratinib. This hypothesis, testing the interaction of an ADC with a TKI, reversible and irreversible, could be evaluated in patient-derived xenografts or other model systems and should be validated prior to a randomized trial. Finally, a small fraction of HER2-nonamplified patients did benefit from T-DM1 + neratinib. Possible explanations, which require further investigation, include a false negative assay, enhanced internalization of T-DM1 in presence of neratinib, or EGFR becoming the driver in patients with loss of HER2-amplification, and inhibition by neratinib [32–34]. We also realize that a low ctDNA fraction could have prevented the detection of ctHER2 amplification.

Gene expression analysis revealed several important observations. First, the level of HER2 RNA expression in TP1 tissues was closely correlated with the response rate to study therapy. Second, non-luminal subtypes had a better response rate than luminal tumors, although this difference was not statistically significant. Third, there was a non-significant association of the 8-gene trastuzumab benefit groups with the rate of responses to study therapy. Fourth, changes in intrinsic subtypes were seen between TP0 and TP1 tissue samples, indicating that these changes may be a result of tumors evolving to become resistant to HP. These results highlight the importance of collecting and monitoring molecular changes in tissue samples as patients move through their treatments.

PIK3CA mutations are a known oncogenic driver in breast cancer and drive therapeutic resistance in multiple HER2-targeted therapies [35]. In the EMILIA trial, patients with PIK3CA mutations, treated with capecitabine + lapatinib were associated with a shorter PFS than were patients with wild-type tumors; however, in patients treated with T-DM1 this was not the case [36]. We likewise see that in patients treated with T-DM1 + neratinib, PIK3CA mutations were not associated with outcomes. However, we cannot rule out the possibility that a subset of patients, refractory to T-DM1 + neratinib with PIK3CA mutations, may be responsive to PIK3CA inhibitors.

## Conclusions

We demonstrate the usefulness of serial assessment of HER2 status in blood and tissue in patients with an initial diagnosis of HER2-positive disease. Loss of HER2 amplification in ctDNA, or the complete loss of ctDNA on treatment with T-DM1 + neratinib, was associated with clinical benefit. Further, we show that many of the patients with short-lived PR or PD were HER2-low in tissue. These patients may be better treated with the recently approved ADC, trastuzumab deruxtecan. We observed that the ADC, T-DM1, plus neratinib, was well tolerated. The combination with an irreversible tyrosine kinase inhibitor with other ADCs warrants investigation.

### Supplementary Information

**Additional file 1**. Additional Patient information and Methodological Details.

**Additional file 2**. All Molecular and Response Data Information for NSABP FB-10 Patients.

## Data Availability

Anonymized individual participant data that underlie the results reported in this article will be available in dbGAP or other publicly available site after publication.
