# Correction: NSABP FB-10: a phase Ib/II trial evaluating ado-trastuzumab emtansine (T-DM1) with neratinib in women with metastatic HER2-positive breast cancer

**DOI:** 10.1186/s13058-024-01833-6

**Published:** 2024-05-24

**Authors:** Samuel A. Jacobs, Ying Wang, Jame Abraham, Huichen Feng, Alberto J. Montero, Corey Lipchik, Melanie Finnigan, Rachel C. Jankowitz, Mohamad A. Salkeni, Sai K. Maley, Shannon L. Puhalla, Fanny Piette, Katie Quinn, Kyle Chang, Rebecca J. Nagy, Carmen J. Allegra, Kelly Vehec, Norman Wolmark, Peter C. Lucas, Ashok Srinivasan, Katherine L. Pogue-Geile

**Affiliations:** 1 NSABP Foundation, Pittsburgh, PA USA; 2 Cleveland Clinic, Taussig Cancer Institute, Weston, Cleveland, OH USA; 3 University Hospitals/Seidman Cancer Center, Case Western Reserve University, Cleveland, OH USA; 4 University of Pittsburgh, Pittsburgh, PA USA; 5 Present Address: University of Pennsylvania Perelman School of Medicine, State College, Philadelphia, PA USA; 6 National Institutes of Health, Washington, DC USA; 7 Present Address: Virginia Cancer Specialists, Fairfax, VA USA; 8 UPMC Hillman Cancer Center, Pittsburgh, PA USA; 9 University of Pittsburgh School of Medicine, Pittsburgh, PA USA; 10 International Drug Development Institute, Louvain-la-Neuve, Belgium; 11 Guardant Health, Redwood City, CA USA; 12 University of Florida Health, Gainesville, FL USA; 13 Department of Laboratory Medicine and Pathology, Mayo Clinic, Rochester, MN USA; 14 Autism Impact Fund, Pittsburgh, PA USA

**Correction: Jacobs et al. Breast Cancer Research (2024) 26:69.**

10.1186/s13058-024-01823-8.

Following the publication of the original article, there were several errors, as follows:

In the abstract, Results section, “95% CI” should be included in the sixth sentence. It should read: “Molecular Response scores were significantly associated with both PFS (HR 0.28, 95% CI 0.09-0.90, P = 0.033) and best response (P = 0.037).”

On page 4, left-hand column: The word “samples,” after the word “two,” was mistakenly removed during typesetting. The correct statement should have been “ … the percent ctDNA change between the two timepoints based on the mean variant allele frequency (VAF) between two samples (mean VAF_2_/mean VAF_1_) -1 ? 100%.”

On page 7, left-hand column, a statement of “0.09-0.90, P = 0.033 using Wilcox test” was duplicated and has been removed.

The original article has been corrected.

