# Supplementary material for: NSABP FB-10: a phase Ib/II trial evaluating ado-trastuzumab emtansine (T-DM1) with neratinib in women with metastatic HER2-positive breast cancer
Source: Breast Cancer Res. 2024 Apr 22;26:69. doi: 10.1186/s13058-024-01823-8 (PMC11036567; doi:10.1186/s13058-024-01823-8)
Supplement: Supplementary file 1 — Additional file 1. Additional Patient information and Methodological Details. [file 13058_2024_1823_MOESM1_ESM.docx]

**Supplementary Information, Tables, and Figures**

**NSABP FB-10: A Phase Ib/ II Trial Evaluating Ado-trastuzumab Emtansine**

**(T-DM1) with Neratinib in Women with Metastatic HER2+ Breast Cancer**

**Evaluation of HER2 status by CLIA data**

The pathologist at our reference laboratory (Magee Women’s Hospital Pathology Department) reviewed the cases in their CLIA facility. HER2 CLIA assays were performed in two separate batches. In the first batch, the frequency of IHC 0 and 1+ was 12% (4/33), which was not considered particularly unexpected given that the concordance rate of HER2 status between CLIA laboratories was only 8.4% in a recent study.^1^ Because the CLIA scoring of HER2 does not require FISH analysis in samples that are scored 0 or 1+, the pathologist followed routine procedure, which did not require a FISH analysis. However, in the second batch of samples, done separately, the frequency of IHC 0 and 1+ was 9/22, or 41%. Due to this unexpectedly high frequency of low IHC 0 and 1+ scores, the pathologist chose to run FISH assays for all the available cases from Batch Two. Within these 9 samples from Batch Two, 8 cases had available tissue. For these 8 cases, 2 failed the FISH assay, 2 were not amplified, and 4 were amplified. We were not able to retest IHC 0 and 1+ samples in Batch One because the blocks were sent back to their respective institutions upon their request. **Supplementary Table S2**). If a sample tested positive for HER2 based on FISH, then it was considered HER2 amplified, regardless of the IHC status.

**Ion Torrent DNA sequencing**

DNA sequencing was performed using a custom Ampli-Seq panel referred to as the NAR panel, amplifying 3,847 amplicons with 94.25% coverage of exons from 117 genes in HER2-activated pathways (**Supplementary Table S1**). The panel was designed using the Thermo Fisher Ion AmpliSeq^TM^ Designer tool ([https://www.ampliseq.com](https://www.ampliseq.com/browse.action)). Libraries were constructed using 10ng of DNA using the Ion AmpliSeq^TM^ kit for Chef DL8. The Ion Chef instrument was used to template and load samples on Ion 550 chips. Up to 32 samples were barcoded, pooled, and sequenced on the S5 sequencer (ThermoFisher) following manufacturer’s instructions.

**HER2 Copy number**

HER2 Copy number was determined using Ion Torrent, NAR Ampliseq data and a tumor-normal pipeline available in the Ion Reporter cloud (<https://ionreporter.thermofisher.com>), provided by ThermoFisher Scientific Inc. The analysis was conducted with a comparison between a tumor sample and a NA12878 DNA sample as the normal control by using our NAR AmpliSeq data.  HER2 Copy numbers of all tumor samples were calculated against the NA12878 reference control. Copy numbers equal to or above a threshold of 4 were filtered out for subsequent analysis.

**Variant calling for Ion Torrent data**

Data from S5 sequencing runs were processed using the Ion Torrent platform pipeline software to generate sequence reads, trim adapter sequences, and remove poor signal-profile reads. Variant calling was generated with the Torrent Suite Software v5.14 with a plug-in “variant caller” program utilizing the somatic-low stringency option. A total of 35,687 variants were detected. The GenomOncology platform was used to annotate and filter these variants with the dbSNP155 dataset. Filtering was undertaken to limit the number of variants. Variants were removed if they were not located in exons or splice junctions, had a coverage depth <300, a quality score of <25, or a variant allele frequency of <5%. Additional variants were removed if they were detected in the blood from FB-10 patients or were included in the dbSNP155 database. However, pathogenic variants included in dbSNP were not removed from our list of variants. Low quality samples were removed from the dataset, which included FB10-042-TP0, FB10-353-TP0, FB10-369-TP0, FB10-390-TP0, FB10-539-TP0, FB10-791-TP0, FB10-870-TP0, FB10-913-TP0, and FB10-963-TP0. After these filters were applied, ~245 variants were identified. No variants were found in the following samples: FB10-071-TP1, FB10-270-TP0, FB10-311-TP0, FB10-388-TP0, FB10-488-TP0, and FB10-963-TP1.

Variants were selected with a VAF of >10% to limit the number of variants detected (**Supplementary Table S5A**). In addition, we also selected variants with a VAF of >5% to maximize the concordance between ctDNA and tissues (**Supplementary** **Table S5B**). The striking observation was that although most of the samples had two or fewer variants, a distinct minority (n=6) had 9 or more variants at an allele frequency of ˂5%. Because formalin fixation can result in C to T and G to A base transitions, we assessed the prevalence of different base changes among these variants.^1^ Selecting variants with a VAF >5%, we found that the nucleotide changes were predominantly C to T or G to A transitions and the distribution of the variants were mostly below 5%. However, when we selected variants with a VAF >10 %, the nucleotide changes and the VAF distribution were more evenly distributed between C to T and G to A (**Supplementary Fig. S1**).

Despite the number of variants that are likely due to formalin fixation, we noticed that there were variants detected at a frequency below 10% that could be of biological relevance (**Supplementary Table S5B**). The ERBB2 variant V777L was detected in both the TP0 and TP1 samples and in the C1D1 ctDNA in a patient whose best response was CR (FB10-605). This observation of a good response in a patient with the V777L mutation is consistent with other studies showing that HER2-positive tumors with this mutation were sensitive to neratinib treatment.

**DNA variants detected in PD and CR patients**

Fourteen variants located in 12 different genes were detected only in PD tumors (**Supplementary Table** **S5A**). To assess whether any of these variants might be clinically relevant, we compared the FB-10 variants to variants described for breast cancer patients in the cBioPortal database. A variant in the ERBB4 gene located at aa 1010 in FB10-440 was also found in one sample in cBioPortal, although the aa change was different (E1010K vs E1010V). The ADAM17-S770L variant in FB10-490 overlapped the S767 frame shift variant in one breast cancer sample. It is unknown whether any of these variants are relevant for the lack of response seen in these patients but could be explored in future studies.

The FOXO3-P174L variant in FB10 in FB10-579 may also be playing a role in the resistance to anti-HER2 therapy. FOXO3 is a transcription factor that upregulates estrogen receptor signaling. Breast cancer cells have been shown to acquire resistance to anti-HER2 therapy via de-repression of the FOXO3 transcription factor in breast cancer cells. FOXO3 de-repression resulted in estrogen receptor activation and/or increased HER3 signaling.^2^ FOXO3 variants adjacent to the FB10 variant have been detected (S173F/Y) in two breast cancers (cBioPortal).

We also detected the ESR1-Y537S variant in FB10-335 TP1 (but not in TP0), which is a well-known variant responsible for resistance to aromatase inhibitors. One could speculate that this variant was selected due to treatment with an aromatase inhibitor. This patient may potentially be responsive to a SERD combination.^3^

Variants are also known to increase the sensitivity to treatment. Two variants in the GRB7 gene (E141D, K304N) were detected in one patient (FB10-988) with a CR. GRB7 plays a pivotal role in transmission of ERBB2 signaling and is often amplified in HER2-amplified tumors due in part to its chromosomal location, which is adjacent to the ERBB2 gene. It is possible that these GRB7 variants increase HER2 signaling and render the cancer cells very dependent upon the HER2 signaling cascade. HER2 and GRB7 are known to physically interact, and this interaction is essential to HER2 signaling. In the HER2-amplified cell line (SKBR3), GRB7 binds tightly to HER2 in such a fashion that many of the ERBB2 tyrosine phosphorylated sites are bound to GRB-7.^4^ This interaction of GRB7 and ERBB2 is essential to transmit and amplify the oncogenic ERBB2 signal and it seems quite likely that neratinib may block the GRB7 and ERBB2 interaction resulting in the loss of HER2 signaling.^2^

Another variant that was detected only in patients with a CR was FOXO1-W189* in patient FB10-916. FOXO1 is also a transcription factor, a central regulator of cellular homeostasis and a tumor suppressor in many different cancers, including breast. FOXO1 is regulated by AKT, which phosphorylates FOXO1 at Thr24, Ser256, and Ser319. Phosphorylation of FOXP3 at Ser256 prevents the translocation of FOXP3 to the nucleus.^5^ The FOXO1-W189* protein may still be able to be translocated to the nucleus. However, it is unclear whether the FOXO1-W189* peptide would have any activity that would result in sensitivity to anti-HER2 therapy.

**Whole transcriptomic analysis**

The phase Ib RNAs were made library-ready via the HTG EdgeSeq system and the HTP whole transcriptome panel, which includes probes which represent most of the human transcriptome (19,398 genes). These library-ready sequences were used as templates to amplify and create barcoded cDNA libraries which were purified, quantitated, and pooled. Each pool included 16 samples and were sequenced with the Ion Torrent sequencing system using the S5 instrument and 550 chips. Samples were normalized to remove batch effects between the two sequencing methods by sva R package. After normalization, Principal Components Analysis (PCA) was applied to remove outliers and lower quality repeated samples. The remaining 48 samples were shown to have good quality, which was demonstrated by the low variance of 6 housekeeping genes (PUM1, RPL13A, B2M, ACTB, GAPDH, TBP) and good correlation of ERBB2- and ESR1-related genes.

**Supplementary Table S1**. Genes included in the custom DNA sequencing panel, NAR

| ACTA1 | FOXO3 | MAP2K2 | PTEN |
| --- | --- | --- | --- |
| ADAM17 | GAB1 | MAP2K4 | PTK2 |
| ADCY1 | GATA3 | MAP3K1 | PTK2B |
| AKT1 | GH1 | MAP4K1 | PTPN11 |
| ARRB1 | GHR | MAPK1 | PTPRR |
| BAD | GNAS | MAPK3 | PXN |
| BRAF | GNB1 | MAPK8 | RAF1 |
| CA12 | GRB2 | MET | RAP1A |
| CARM1 | GRB7 | MKNK1 | RAP1B |
| CASP9 | GRIP1 | MKNK2 | RAPGEF1 |
| CDKN1B | HCK | MTOR | RASA1 |
| CHUK | HGF | MYC | RELA |
| CRK | HRAS | NFKB1 | RPS6 |
| CRKL | HRG | NFKBIA | RPS6KA1 |
| CSNK2A1 | HSP90AA1 | NGF | RPS6KA5 |
| DNM1 | IGF1 | NGFR | RPS6KB1 |
| DOCK1 | IGF1R | NRAS | SHC1 |
| EGF | IKBKB | PAK1 | SOS1 |
| EGFR | IKBKG | PDGFRA | SRC |
| EIF2B5 | IL6 | PDK2 | SRF |
| EIF2S1 | IL6R | PDPK1 | STAT1 |
| EIF2S2 | IL6ST | PIK3CA | STAT2 |
| EIF2S3 | INPPL1 | PIK3CG | STAT3 |
| ERBB2 | ITGA1 | PIK3R1 | STAT4 |
| ERBB3 | ITGB1 | PLCB1 | STAT5A |
| ERBB4 | JAK1 | PLCG1 | STAT5B |
| ESR1 | JUN | PPP2CA | STAT6 |
| FASLG | KRAS | PRKCA | TGFBR1 |
| FOXO1 | MAP2K1 | PRKCB | TOP2A |
|  |  |  | YWHAH |

**Supplementary Table S2.** Equivocal IHC0 and 1+ in Batch 2: NSABP FB-10

| **Patient ID** | **HER2 IHC TP0** | **HER2 FISH TP0** | **HER2 IHC/FISH TP0** |
| --- | --- | --- | --- |
| FB10-189 | 1+ | not amp | not amp |
| FB10-635 | 1+ | not amp | not amp |
| FB10-256 | 1+ | no slide for FISH | not determined |
| FB10-042 | 0 | failed | not determined |
| FB10-390 | 1+ | failed | not determined |
| FB10-388 | 0 | Amplified | Amplified |
| FB10-870 | 0 | Amplified | Amplified |
| FB10-270 | 1+ | Amplified | Amplified |
| FB10-488 | 1+ | Amplified | Amplified |

**Supplementary Table S3.** Patient characteristics in Phase Ib and Phase II: NSABP FB-10

| **Characteristic** | **Phase Ib (n=27)** | **Phase II (n=22)** |
| --- | --- | --- |
| Age  Median  Range | 48  23-69 | 58  32-70 |
| Performance status  0  1 | 20  7 | 10  12 |
| Hormonal status  ER or PR +  ER and PR - | 15  12 | 13  9 |
| Brain metastases  Yes  No | 6  21 | NA  NA |
| Disease burden  Single organ  Multiple organs | 6  12 | NA  NA |

**Supplemental Table S4:** Efficacy by ctDNA HER2 status at C1D1: NSABP FB-10

| **Response** | **All pts with ctHER2 data**  **N=38*** | **ctHER2-amp n=20** | **ctHER2-**  **no amp**  **n=17** | **HER2-indeterminant n=5** |
| --- | --- | --- | --- | --- |
| CR/PR | 18 (47%) | 11 (55%) | 7 (41%) | 1 (20%) |
| SD | 2 (5.3%) | 1 (5%) | 1 (5.9%) | 1 (20%) |
| CBR | 20 (53%) | 12 (60%) | 8 (47%) | 0 |
| Mean DOR, days | ND | 457 | 131 | ND |

CR=complete response; PR=partial response; CBR=clinical best response; SD=stable disease; DOR=duration of response, ND=not determined.

*Total patients here is 38, whereas for the Molecular Response scores, the total is 37, because the blood sample that was drawn was not eligible for Guardant Molecular Response evaluation because the patient progressed before the blood was drawn but values for ctDNA were determined.

**Supplementary Table S5.** All Molecular and Response Data Information: NSABP FB-10

Included separately as an excel file.

**Supplementary Table S6.** Intrinsic Subtypes and Responses in NSABP FB-10 Patients

**TP0* (34 samples, 31 with outcomes)**

Basal HER2 LumA LumB Normal DLT

Total 4 19 1 8 2 3

% 12% 56% 2.9% 24% 5.9% 8.8%

**Outcomes**

CR/PR 1 10 0 3 2

PD 1 8 1 4 0

SD 0 1 0 0 0

DLT 2 0 0 1 0

**TP1 (13 samples, 13 with outcomes)**

Basal HER2 LumA LumB Normal

Total 3 6 1 3 0

% 23% 46% 8% 23% 0%

**Outcomes**

CR/PR 2 3 1 0 0

PD 1 2 0 2 0

SD 0 1 0 1 0

DLT, Dose-limiting toxicity; CR/PR=complete response/partial response; PD=partial response.

**Supplementary Fig. S1**

**
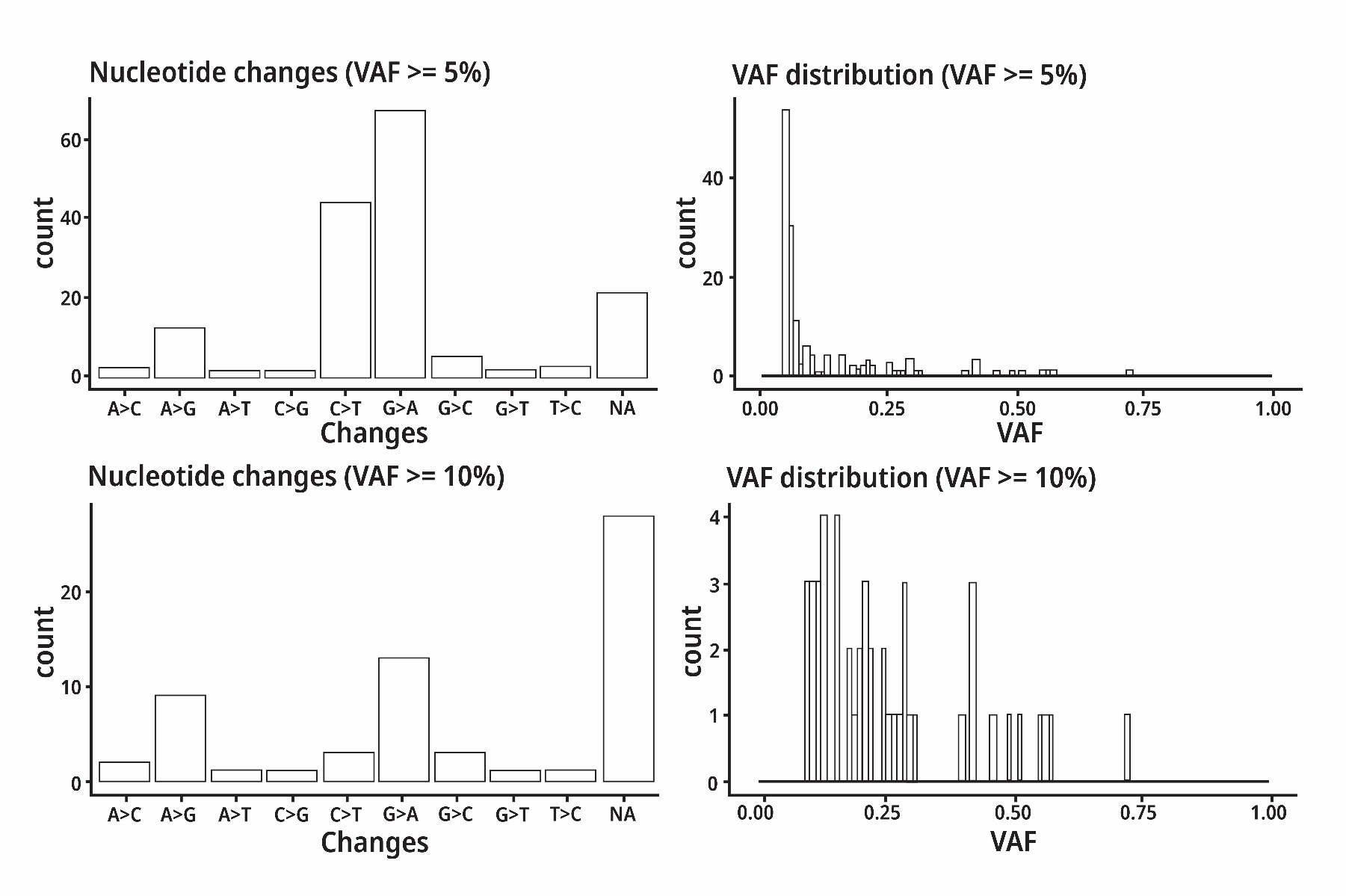
**

**Supplementary Fig. S2**


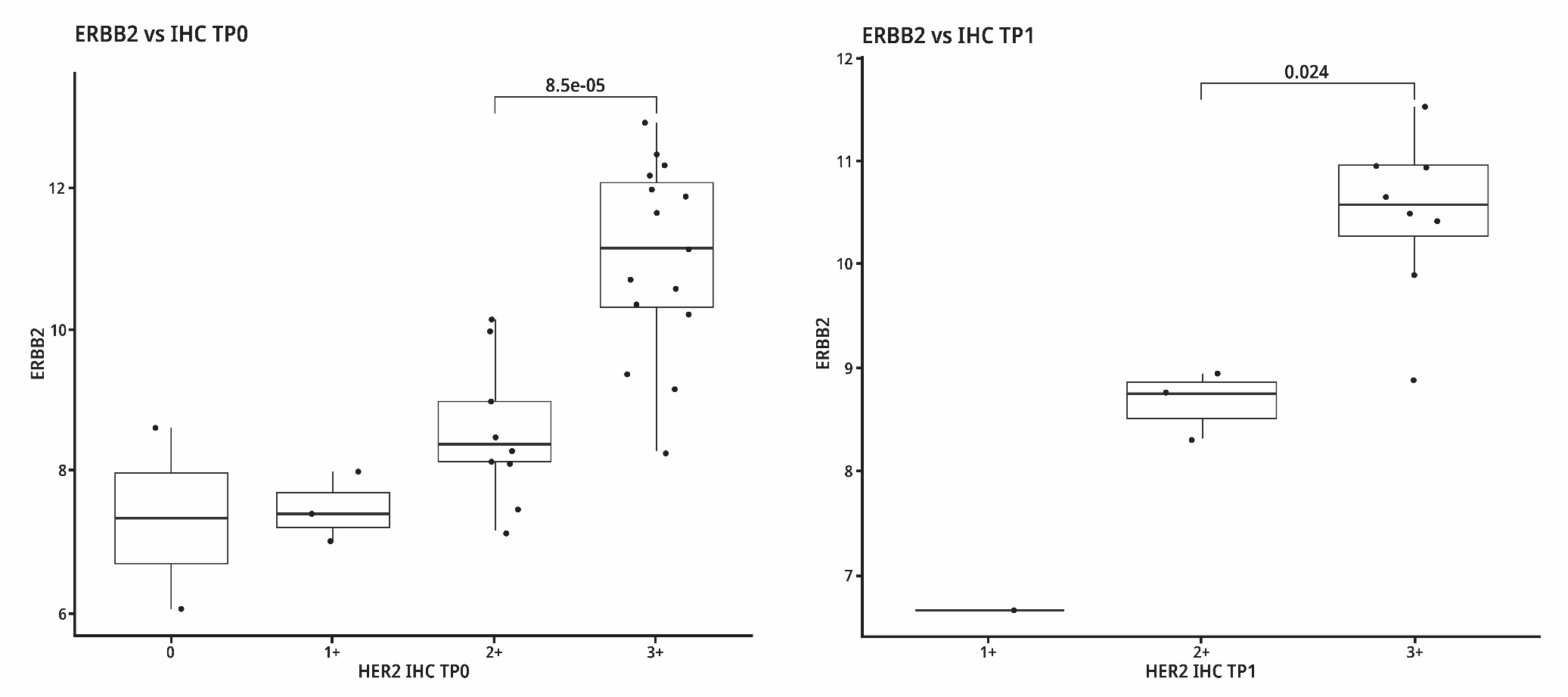


**Supplementary Fig. S3**


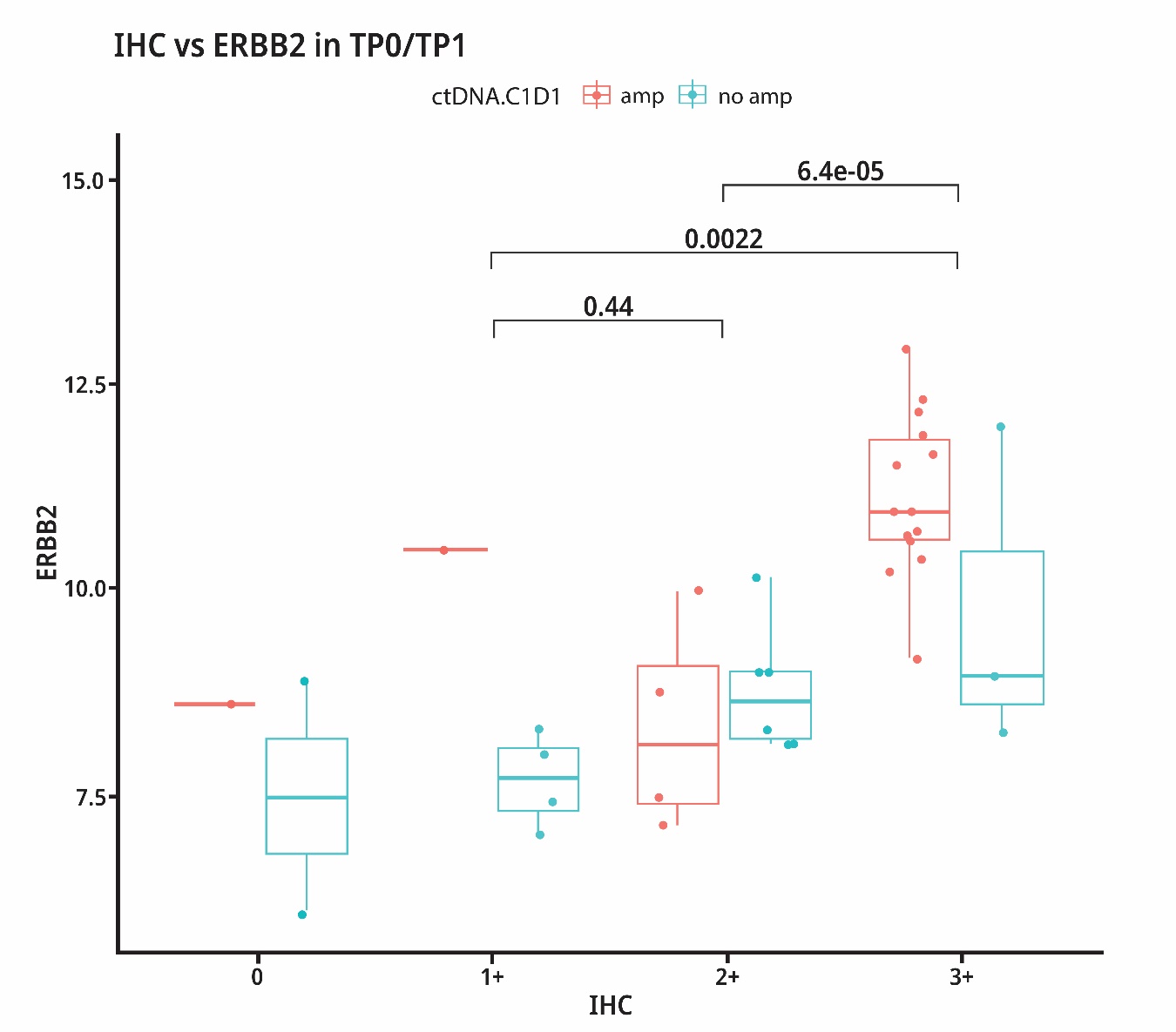


**SUPPLEMENTARY REFERENCES**

1. Mathieson W, Thomas GA. Why formalin-fixed, paraffin-embedded biospecimens must be used in genomic medicine: An evidence-based review and conclusion. J Histochem Cytochem; 68:543-552, 2020

<https://pubmed.ncbi.nlm.nih.gov/32697619/>

2. Nencioni A, Cea M, Garuti A, et al. Grb7 upregulation is a molecular adaptation to HER2 signaling inhibition due to removal of Akt-mediated gene repression. PLoS One; 5:e9024, 2010

<https://pubmed.ncbi.nlm.nih.gov/20126311/>

3. Toy W, Weir H, Razavi P, et al. Activating ESR1 mutations differentially affect the efficacy of ER antagonists. Cancer Discov; 7:277-287, 2017

<https://pubmed.ncbi.nlm.nih.gov/27986707/>

4. Stein D, Wu J, Fuqua SA, et al. The SH2 domain protein GRB-7 is co-amplified, overexpressed and in a tight complex with HER2 in breast cancer. EMBO J; 13:1331-1340, 1994

<https://pubmed.ncbi.nlm.nih.gov/7907978/>

5. Yadav RK, Chauhan AS, Zhuang L, Gan B. FoxO transcription factors in cancer metabolism. Semin Cancer Biol; 50:65-76, 2018

<https://pubmed.ncbi.nlm.nih.gov/29309929/>
